# Rumen modulatory effect of thyme, clove and peppermint oils *in vitro* using buffalo rumen liquor

**DOI:** 10.14202/vetworld.2015.203-207

**Published:** 2015-02-23

**Authors:** Debashis Roy, S. K. Tomar, Vinod Kumar

**Affiliations:** 1 Department of Animal Nutrition, College of Veterinary Science and Animal Husbandry, Uttar Pradesh Pandit Deen Dayal Upadhyaya Pashu Chikitsa Vigyan Vishwavidyalaya Evam Go Anusandhan Sansthan (DUVASU), Mathura, Uttar Pradesh, India; 2 Dairy Cattle Nutrition Division, National Dairy Research Institute, Karnal, India

**Keywords:** ammonia nitrogen, essential oil, rumen fermentation, methane, wheat straw

## Abstract

**Aim:**

The present study was conducted to examine the rumen modulatory effect of thyme, clove and peppermint oils on rumen fermentation pattern *in vitro* using roughage based diet.

**Materials and Methods:**

Thyme, clove and peppermint oils were tested at concentration of 0, 30, 300 and 600 mg/l (ppm) of total culture fluid using *in vitro* gas production technique in wheat straw based diet (concentrate: Wheat straw 50:50). Different *in vitro* parameters e.g., total gas production, methane production, nutrient degradability, volatile fatty acid (VFA) production and ammonia nitrogen concentration were studied using buffalo rumen liquor.

**Results:**

Thyme oil at higher dose level (600 ppm) reduced (p<0.05) total gas production, feed degradability and ammonia nitrogen (NH_3_-N) concentration whereas total VFA concentration was significantly lower (p>0.05) in 300 and 600 ppm dose levels. 600 ppm dose level of clove oil reduced (p<0.05) total gas production, feed degradability, total VFA and acetate to propionate ratio. Methane production was significantly reduced (p<0.05) in 300 and 600 ppm dose levels of clove and peppermint oil.

**Conclusion:**

Right combination of these essential oils may prove to enhance performance of animals by reducing methane production and inhibiting protein degradation in rumen.

## Introduction

Appearance of residue and resistant strain of bacteria, acceptance of antibiotics as growth promoter and rumen fermentation modulator is gradually reducing or totally banned in some countries. Essential oils (EOs) are emerging as a potent alternative of feed additive due to its natural availability. They are plant secondary metabolites and present in some spice, condiments and different parts of plants. The word “EOs” has come from “essence,” which means sweet fragrance. Thymol, carvacol, eugenol, limonene, allicin, diallyldisulfide are the active compounds present in the Eos, which are responsible for the odour.

Some EOs has antimicrobial activities [1] and is currently considered safe for human and animal consumption, and termed as generally recognized as safe or GRAS [2]. Several workers reported promising effect of different EOs in modulating rumen function *in vitro* [3-5]. Potentiality of different EOs and their combination have also been studied in different ruminant species [6-9] where most of the workers reported positive results due to supplementation. A total of 6-8% of Gross energy of the feed is reported to be lost by methane. In addition, ruminant livestock contributes about 80 million metric tons of methane annually, accounting for about 28% of global methane emissions from human-related activities [10].

However, most of the studies were conducted using some patented EOs or their combination. Due to paucity of studies of effect of different EOs on rumen fermentation parameters *in vitro* in Indian condition, present study was conducted to observe the effect of thyme, clove and peppermint oil on rumen fermentation pattern *in vitro* using buffalo rumen liquor in straw based diet.

## Materials and Methods

### Ethical approval

Ethical approval for fistulation of buffalo bull and collection of rumen liquor from those bulls was taken from Institutional Animal Ethics Committee constituted as per the article no. 13 of the CPCSEA rules laid down by Government of India.

### Animal feeding and sample analysis

Rumen liquor was collected from two donors fistulated buffalo bull maintained by the herd of National Dairy Research Institute, Karnal, Haryana, India. Fistulation of buffalo bull was performed by surgeon as per regulation of Institutional Animal Ethics Committee. Rumen liquor was collected before morning feeding into a pre-warmed thermo-flask and brought to the laboratory. Donor animals were fed on wheat straw and concentrate based diet (3.0 kg concentrate mixture and wheat straw ad libitum).

### Source of EOs

Thyme, clove and peppermint oils standards were supplied by Sigma-Aldrich chemicals Pvt. Limited (USA). Each EO was diluted to prepare three dilutions, i.e., 30, 300 and 600 ppm in 30 ml of incubation medium from standard supplied by Sigma-Aldrich chemicals Pvt. Limited.

### Proximate analyses of substrate

Diet samples were collected and analyzed for dry matter (DM) (ID number 930.15), organic matter (OM) and ash (ID number 942.05) and crude protein (N × 6.25, ID number 954.01), ether extract (ID number 920.39) contents, in accordance with the AOAC [11]. The neutral detergent fiber and acid detergent fiber analysis were based on the procedures described by Van Soest and Robertson [12].

### *In vitro* gas and methane production

*In vitro* gas production was analyzed following the methodology adapted by Menke and Steingass [13]. 0.2 g of wheat straw (*Triticum aestivum*) and concentrate mixture (50:50 ratio) were used as substrates and mulberry leaves (*Morus alba*) as an internal standard. Concentrate mixture was consist of maize 33%, groundnut cake (oiled) 21%, mustard oil cake (oiled) 12%, wheat bran 20%, deoiled rice bran 11%, mineral mixture 2% and common salt 1%. Chemical composition of concentrate mixture and wheat straw used in the experiment is presented in Table-1. Gas production in blank (containing inoculum) and test syringe (containing inoculum and substrate) were measured after 24 h. The gas produced in standard syringe (containing mulberry leaves) was used to check the day to day variation of inoculum. Methane was estimated by using Nucon-5700 gas chromatograph (GC) equipped with flame ionization detector and stainless steel column packed with Porapak-Q. The standard gas used for methane estimation (Spantech Calibration gas, Surrey, England) composed of 50% methane and 50% CO_2_. The methane produced from the substrate during 24 h incubation was corrected for the blank values. The volume of CH_4_ (ml) produced was calculated as follows;

**Table-1:** Chemical compositions (%DM basis) of substrate (concentrate mixture and wheat straw).

	DM	OM	Crude protein	Ether extract	Crude fiber	NFE	Total Ash	NDF	ADF
Concentrate mixture	90.1	92.8	21.5	3.1	6.6	61.5	7.7	30.7	12.1
Wheat straw	90.4	89.4	3.6	0.9	38.1	47.8	11.3	83.6	50.1

NFE=Nitrogen free extract, NDF=Neutral detergent fibre, ADF=Acid detergent fibre, OM=Organic matter, DM=Dry matter

Methane production (ml) = Total gas produced (ml) × % methane in the sample.

### In vitro degradability of feed, ammonia- N (NH_3_-N) and volatile fatty acid (VFA) estimation

True DM and OM degradability was determined by transferring the content of each syringe quantitatively into a centrifuge tube and centrifuged at 5000 rpm for 15 min. True degradability was estimated after 24 h incubation as per the method outlined by Goering and Soest [14]. Truly degradable OM in rumen (TDOMR) was estimated by subtracting incubated OM with substrate recovered as residue after ND solution treatment. However, partitioning factor was calculated as the ratio of TDOMR (mg) to gas volume (ml) produced during 24 h incubation. For NH_3_-N estimation, 5 ml of supernatant was mixed with 2 ml of 1 N NaOH and steam distilled using KEL PLUS - N analyzer (Pelican, India). Released NH_3_-N was trapped in boric acid solution having mixed indicator and titrated against 0.01N H_2_SO_4_ [15]. Total VFA concentration was estimated as per method described by Barnett and Reid [16]. For the estimation of individual VFAs, 5 ml of supernatant was treated with 1 ml meta-phosphoric acid (25%) and kept overnight at 4°C. Different VFA’s of the samples were identified using GC (Nucon 5700, India) on the basis of their retention time and their concentration (mmol) was calculated by comparing the retention time as well as the peak area of standards after deducting the corresponding blank values [17].

### Statistical analysis

The generated data were statistically analyzed by analysis of variance considering dose of EOs as factor using the general linear model procedure (univariate) according to *Yij = μ + Di+ e_ij_* with *Yij* as the studied parameter (j^th^ observation on the i^th^ treatment), µ as population mean, *Di* as effect of the EOs (effect of the i^th^ group) and eij as residual error associated with the ij^th^ observation. Means were compared using Tukey’s HSD test [18]. Significant differences were accepted if p>0.05. All statistical analyses were performed using SPSS 19 [19].

## Results and Discussion

Proximate principle and fibre fraction of concentrate mixture and wheat straw used in the experiment is presented in Table-1. The values of nutrient composition are comparable with the findings of Ranjhan [20] and Ayyappan *et al*. [21]. 600 ppm dose level of thyme and clove oil resulted in a decrease (p<0.05) in gas production, OM and DM degradability (Tables-2 and -3). Thyme oil at 300 and 600 ppm reduced total VFA concentration by 20.2 and 40.4%, respectively (Table-2), compared with control. Whereas, relative to control, the concentration of total VFA was reduced by 14.7% at 600 ppm dose rate of clove oil (Table-3). The main active compounds of thyme and clove oil are thymol and eugenol, respectively. Overall decrease in rumen fermentation in high dose level of thyme and clove oil may be due to their active principles (thymol and eugenol, respectively), which are more effective antimicrobials in comparison with other non-phenolic secondary plant metabolites because of the presence of a hydroxyl group in the phenolic structure and results into loss of integrity of bacterial cell membrane which ultimately resulted in reduction in glucose uptake by bacteria [22-25]. Reduction in total VFA concentration was also reported other workers using thyme and clove oil *in vitro* [26,27]. Peppermint oil did not adversely affect feed degradability in all the dose level, which is similar to the findings of Craig [28]. Methane concentration was reduced by 16.6 and 18.1% in 300 and 600 ppm of peppermint oil compared with control, whereas 12.3 and 26.6% reduction in methane concentration was found in clove oil in above dose levels, respectively (Tables-3 and -4). Present finding corroborate the observation of Agarwal *et al*. [29] who reported reduction in methane production by 19.9%, 46.0% and 75.6% at 0.33, 1.0 and 2.0 μl of peppermint oil. It may be postulated that the reduction of methane was due to the reduction of total protozoa [30] by menthol (active principle of peppermint oil). Similar to our finding, Patra *et al*. [31] reported inhibition of methane production with reduced digestibility of the feed *in vitro* with clove oil. The concentration of NH_3_-N was reduced (p<0.05) in high dose level (600 ppm) of thyme oil (Table-2). Reduction (p<0.05) in acetate to propionate ratio was observed in high (600 ppm) dose level of clove oil. Reduction in concentration of NH_3_-N is suggesting the potentiality of thymol in inhibiting deamination and corroborating the study of past workers [26,32] who found a similar result when incubated rumen fluid with thymol *in vitro*. Evans and Martin [33] in their study in pure culture reported that thymol affected the energy metabolism of two major rumen deaminating bacteria, *Streptococcus bovis* and *Selenomonas ruminantium*. Corroborating the study of Busquet *et al*. [27], present study also reported reduction of acetate propionate ratio in high dose level of clove oil which could be a good indicator of simultaneous methane reduction in the rumen [34].

**Table-2:** Effect of different levels of thyme oil on *in vitro* fermentation parameters (mean±SD) in wheat straw based (concentrate: Roughage=50:50) diet.

Attributes	Control	30 ppm	300 ppm	600 ppm	p value
Total gas (ml/24 h/200 mg substrate)	36.00^a^±1.65	35.50^a^±1.54	34.83^a^±1.75	23.16^b^±1.30	<0.01
True OM degradability (%)	65.13^a^±2.17	62.08^a^±2.55	61.90^a^±3.65	51.30^b^±5.11	<0.01
True DM degradability (%)	67.16^a^±2.84	65.33^a^±3.67	66.50^a^±1.73	55.16^b^±4.64	<0.01
Methane (ml/24 h)	8.10±3.48	10.11±2.25	10.67±1.82	7.07±1.21	>0.05
Methane (ml/g OMD)	67.08±28.86	87.73±19.57	93.00±15.99	74.33±12.75	>0.05
Methane (ml/g DMD)	60.33±25.95	77.42±17.26	80.28±13.79	64.10±10.99	>0.05
Ammonia nitrogen (mg/100 ml)	32.28^a^±1.80	31.42^a^±1.54	27.99^ab^±1.93	24.90^b^±3.17	0.01
Total VFAs (mmol/100 ml)	51.3^a^±1.87	48.27^a^±1.85	40.94^b^±1.38	30.59^c^±1.96	0.01
VFA (molar proportion)					
Acetate	56.28±2.92	57.91±3.21	58.23±10.68	56.24±3.47	>0.05
Propionate	22.30±1.57	20.32±1.92	20.37±1.26	21.65±0.89	>0.05
Butyrate	6.37±1.15	6.47±1.14	7.77±0.73	10.18±3.22	>0.05
Partitioning factor	3.35^a^±0.14	3.24^a^±0.10	3.33^ab^±0.45	4.10^b^±0.33	0.02

Different superscript a, b, c in a row differ significantly (p<0.05). OM=Organic matter, DM=Dry matter, OMD=Organic matter digested, DMD=Dry matter digested, VFA=Volatile fatty acid

**Table-3:** Effect of different levels of clove oil on *in vitro* fermentation parameters (mean±SD) in wheat straw based (Concentrate: Roughage=50:50) diet.

Attributes	Control	30 ppm	300 ppm	600 ppm	p value
Total gas (ml/24 h/200 mg substrate)	41.33^a^±1.52	41.20^a^±3.86	37.33^ab^±2.36	29.33^b^±5.01	<0.01
True OM degradability (%)	68.72^a^±3.27	69.98^a^±1.71	64.05^ab^±3.41	59.56^b^±2.84	<0.01
True DM degradability (%)	70.16^a^±1.44	71.66^a^±0.76	63.83^ab^±5.33	59.16^b^±3.24	<0.01
Methane (ml/24 h)	12.38^a^±0.48	12.80^a^±0.19	10.86^b^±0.92	9.09^c^±0.57	<0.01
Methane (ml/g OMD)	99.22^a^±3.97	99.67^a^±1.42	95.17^ab^±8.14	86.99^b^±5.46	0.03
Methane (ml/g DMD)	88.23^a^±3.52	89.34^a^±1.28	85.09^ab^±7.27	76.81^b^±4.81	0.02
Ammonia nitrogen (mg/100 ml)	18.89±1.75	17.34±0.54	17.51±0.43	18.63±2.22	>0.05
Total VFAs (mmol)	47.4^a^±1.45	47.62^a^±0.56	46.30^a^±0.72	40.44^b^±0.69	0.04
VFA (molar proportion)					
Acetate	52.13^a^±1.07	50.41^a^±0.65	49.80^a^±1.82	42.73^b^±1.24	0.04
Propionate	20.41^a^±0.48	21.85^a^±1.10	22.52^a^±1.50	25.26^b^±0.60	0.03
Butyrate	8.46±0.54	8.74±1.05	7.69±1.06	9.00±1.53	>0.05
Partitioning factor	3.08±0.05	3.17±0.28	3.18±0.23	3.82±0.54	>0.05

Different superscript a, b, c in a row differ significantly (p<0.05). OM=Organic matter, DM=Dry matter, OMD=Organic matter digested, DMD=Dry matter digested, VFA=Volatile fatty acid, SD=Standard deviation

**Table-4:** Effect of different levels of peppermint oil on *in vitro* fermentation parameters (mean±SD) in wheat straw based (Concentrate: Roughage=50:50) diet.

Attributes	Control	30 ppm	300 ppm	600 ppm	p value
Total gas (ml/24 h/200 mg substrate)	36.83±1.59	34.83±2.87	31.33±6.65	37.66±3.05	>0.05
True OM degradability (%)	47.70±3.76	48.57±2.031	50.27±3.29	50.22±2.42	>0.05
True DM degradability (%)	48.16±1.83	50.00±1.29	53.16±3.03	53.00±1.75	>0.05
Methane (ml/24 h)	12.46a±1.40	11.21ab±0.19	10.35b±0.02	10.21b±0.87	<0.01
Methane (ml/g OMD)	144.12a±0.72	130.22ab±0.33	115.38b±0.78	113.94b±0.62	<0.01
Methane (ml/g DMD)	128.36a±1.30	113.10ab±1.54	96.34b±1.06	95.32b±1.00	<0.01
Ammonia nitrogen (mg/100 ml)	24.06±1.37	24.16±2.18	25.18±1.13	22.76±1.56	>0.05
Total VFAs (mmol)	38.57±3.85	39.96±1.40	44.3±5.22	44.82±5.01	>0.05
VFA (molar proportion)					
Acetate	57.00±5.67	61.23±3.05	55.03±4.30	55.70±4.62	>0.05
Propionate	21.82±3.92	20.25±2.08	22.05±3.21	25.58±3.59	>0.05
Butyrate	8.54±1.07	9.43±2.31	11.51±3.32	11.55±1.66	>0.05
Partitioning factor	2.33a±0.12	2.51a±0.26	2.89b±0.17	2.40a±0.21	0.02

Different superscript a, b, c in a row differ significantly (p<0.05). OM=Organic matter, DM=Dry matter, OMD=Organic matter digested, DMD=Dry matter digested, VFA=Volatile fatty acid

## Conclusion

Higher levels of tested EOs were detrimental to rumen microbes reflecting reduction in total gas production and degradability of feed. Clove and peppermint oil at higher dose rate reduce methane production. Concentration of NH_3_-N was decreased at 600 ppm dose rate of thyme oil due to its selective inhibition of certain deaminating bacteria that may enhance the bypass activity of feed protein. Judicial selection and a careful combination of these EOs allows manipulation of rumen fermentation in a favorable direction.

## Authors’ Contributions

DR planned and carried out research work to compare different EOs *in vitro* for his Ph.D. thesis programme in collaboration with advisory members and guide SKT. VK helped DR in setting overall *in vitro* experiment. All authors participated in draft and revision of the manuscript. All authors read and approved the final manuscript.

## References

[1] Babu A.J, Rupa Sundari A, Indumathi J, Srujan R.V.N, Sravanthi M (2011). Study on the antimicrobial activity and minimum inhibitory concentration of essential oils of spices. Vet. World.

[2] FDA (2004). Freedom of Information Summary. Supplemental New Animal Drug Application. NADA 095-735. Monensin Sodium (RUMENSIN 80):Type A Medicated Article for Dairy Cattle.

[3] Lin B, Lu Y, Wang J.H, Liang Q, Liu J.X (2012). The effects of combined essential oils along with fumarate on rumen fermentation and methane production *in vitro*. J. Anim. Feed Sci.

[4] Lin B, Wang J.H, Lu Y, Liang Q, Liu J.X (2013). *In vitro* rumen fermentation and methane production are influenced by active components of essential oils combined with fumarate. J. Anim. Physiol. Anim. Nutr.

[5] Jahani-Azizabadi H, Danesh Mesgaran M, Vakili A.R, Rezayazdi K, Hashemi M (2011). Effect of various medicinal plant essential oils obtained from semi-arid climate on rumen fermentation characteristics of a high forage diet using *in vitro* batch culture. Afr. J. Microbiol. Res.

[6] Tager L.R, Krause K.M (2011). Effects of essential oils on rumen fermentation, milk production, and feeding behavior in lactating dairy cows. J. Dairy Sci.

[7] Tassoul M.D, Shaver R.D (2009). Effect of a mixture of supplemental dietary plant essential oils on performance of periparturient and early lactation dairy cows. J. Dairy Sci.

[8] Özdoğan M, Önenç S.S, Önenç A (2011). Fattening performance, blood parameters and slaughter traits of Karya lambs consuming blend of essential oil compounds. Afr. J. Biotechnol.

[9] Meyer N.F, Erickson G.E, Klopfenstein T.J, Greenquist M.A, Luebbe M.K, Williams P, Engstrom M.A (2009). Effect of essential oils, tylosin and monensin on finishing steer performance, carcass characteristics, liver abscesses, ruminal fermentation and digestibility. J. Anim. Sci.

[10] US-EPA (2007). http://www.epa.gov/rlep/faq.html.

[11] AOAC (2000). Official Methods of Analysis.

[12] Van Soest P.J, Robertson J.B, James W.P.T, Theander O (1981). The detergent system of analysis and its application to human food. The Analysis of Dietary Fiber in Foods.

[13] Menke K.H, Steingass H (1988). Estimation of energetic feed value obtained by chemical analysis and *in vitro* gas production using rumen fluid. Anim. Res. Dev.

[14] Goering H.K, Van Soest P.J (1970). Forage Fibre Analysis. (Apparatus, Reagents, Procedures, and Some Applications). Agricultural Handbook 379.

[15] Alexander G, Singh B, Sahoo A, Bhat T (2008). *In vitro* screening of plant extracts to enhance the efficiency of utilization of energy and nitrogen in ruminant diets. Anim. Feed Sci. Technol.

[16] Barnett J.G, Reid R.L (1957). Studies on the production of volatile fatty acids from grass by rumen liquor in an artificial rumen. The volatile fatty acid production from grass. J. Agric. Sci. Camb.

[17] Erwin E.S, Macro G.A, Emery E.M (1961). Volatile fatty acid analysis of blood and rumen fluid by gas chromatography. J. Dairy Sci.

[18] Snedecor G.W, Cochran W.G (1994). Statistical Methods. Iowa State University Press.

[19] SPSS (2010). Statistical Packages for Social Sciences, IBM SPSS 19 Statistics.

[20] Ranjhan S.K (1998). Nutrient Requirements of Livestock and Poultry.

[21] Ayyappan K, Tomar S.K (2006). Evaluation of faecal inoculum for estimating digestibility of feedstuffs. Indian J. Anim. Nutr.

[22] Devi K.P, Nisha S.A, Sakthivel R, Pandian S.K (2010). Eugenol (an essential oil of clove) acts as an antibacterial agent against *Salmonella typhi* by disrupting the cellular membrane. J. Ethnopharmacol.

[23] Pei R.S, Zhou F, Ji B.P, Xu J (2009). Evaluation of combined antibacterial effects of eugenol, cinnamaldehyde, thymol, and carvacrol against *E. coli* with an improved method. J. Food Sci.

[24] Bassolé I.H.N, Juliani H.R (2012). Essential oils in combination and their antimicrobial properties. Molecules.

[25] Ultee A, Bennik M.H.J, Moezelaar R (2002). The phenolic hydroxyl group of carvacrol is essential for action against the food-borne pathogen *Bacillus cereus*. Appl. Environ. Microbiol.

[26] Castillejos L, Calsamiglia S, Ferret A (2006). Effect of essential oils active compounds on rumen microbial fermentation and nutrient flow in *in vitro* systems. J. Dairy Sci.

[27] Busquet M, Calsamiglia S, Ferret A, Kamel C (2006). Plant extracts affect *in vitro* rumen microbial fermentation. J. Dairy Sci.

[28] Craig W.J (1999). Health-promoting properties of common herbs. Am. J. Clin. Nutr.

[29] Agarwal N, Shekhar C, Kumar R, Chaudhary L.C, Kamra D.N (2009). Effect of peppermint (*Mentha piperita*) oil on *in vitro* methanogenesis and fermentation of feed with buffalo rumen liquor. Anim. Feed Sci. Technol.

[30] Wallace R.J, McEwan N.R, McIntosh F.M, Newbold C.J (2002). Natural products as manipulators of rumen fermentation. Asian Aust. J Anim.

[31] Patra A.K, Kamra D.N, Agarwal N, Soliva C.R, Takahashi J, Kreuzer M (2005). Effect of spices on rumen fermentation, methanogenesis and protozoa counts in *in vitro* gas production test. Proceedings of the 2^nd^ International Conference of Greenhouse Gases and Animal Agriculture.

[32] Broderick G.A, Balthrop J.E (1979). Chemical inhibition of amino acid deamination by ruminal microbes *in vitro*. J. Anim. Sci.

[33] Evans J.D, Martin S.A (2000). Effects of thymol on ruminal microorganisms. Curr. Microbiol.

[34] Yasuo K (2010). Abatement of methane production from ruminants:Trends in the manipulation of rumen fermentation. Asian Aust. J Anim.

